# Assessing nocturnal scratch with actigraphy in atopic dermatitis patients

**DOI:** 10.1038/s41746-023-00821-y

**Published:** 2023-04-26

**Authors:** Ju Ji, Jordan Venderley, Hui Zhang, Mengjue Lei, Guangchen Ruan, Neel Patel, Yu-Min Chung, Regan Giesting, Leah Miller

**Affiliations:** Eli Lilly & Company, INc., Indianapolis, IN USA

**Keywords:** Autoimmune diseases, Biomarkers, Statistics, Information technology

## Abstract

Nocturnal scratch is one major factor leading to impaired quality of life in atopic dermatitis (AD) patients. Therefore, objectively quantifying nocturnal scratch events aids in assessing the disease state, treatment effect, and AD patients’ quality of life. In this paper, we describe the use of actigraphy, highly predictive topological features, and a model-ensembling approach to develop an assessment of nocturnal scratch events by measuring scratch duration and intensity. Our assessment is tested in a clinical setting against the ground truth obtained from video recordings. The new approach addresses unmet challenges in existing studies, such as the lack of generalizability to real-world applications, the failure to capture finger scratches, and the limitations in the evaluation due to imbalanced data in the current literature. Furthermore, the performance evaluation shows agreement between derived digital endpoints and the video annotation ground truth, as well as patient-reported outcomes, which demonstrated the validity of the new assessment of nocturnal scratch.

## Introduction

Most atopic dermatitis (AD) patients experience itching more often and severely in the evening or nighttime than during the day^1,2^. Sleep disturbance is a concern for AD patients, and itch-related scratch is a major cause. Arousal does not occur prior to itching but ensues after scratch^3,4^. Studies show that itching and scratching are not synonymous concepts, and generally, scratching is not required for mild itch^2^. Patient-reported outcome (PRO) assessments commonly used in trials do not explicitly reflect nocturnal scratches. Furthermore, subjective measures are limited in accurately reflecting the true scratch severity, especially when patients are unconsciously experiencing discomfort that leads to being awake. Therefore, objectively evaluating and quantifying nocturnal scratches can bring additional benefits in understanding the patient’s disease state and life quality. Our study aims to objectively track the duration and intensity of nocturnal scratches for AD patients using wrist-worn actigraphy devices.

**Fig. 1:**
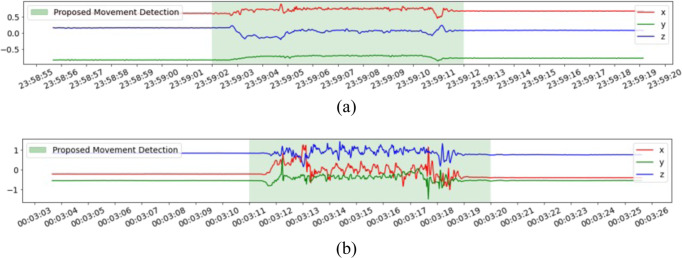
Finger scratch and hand scratch events. **a** A finger scratch event (low-intensity motion pattern). **b** A hand scratch event.

Many recent studies leverage wearable sensor technologies to objectively measure and assess the health-related life quality of patients across various therapeutic areas. These studies demonstrate the benefit of actigraphy devices as cost-effective, noninvasive, and user-friendly ways of enabling proactive personal health management, continuous health monitoring, early detection of symptoms, and context awareness as healthcare costs increase^5–8^. Many of these wearable sensor studies explore nocturnal scratch detection from wrist-worn actigraphy or smartwatch applications. Yang et al.^9^ provide a systematic review of studies published before 2021 to compare methods of measuring scratch objectively and pointed out the large variability in performance and limited evaluation of specificity due to imbalanced data. In addition, lack of generalizability and failure to capture finger scratches would bring additional errors. Figure 1 shows finger and hand scratch examples.

The generalizability of nocturnal scratch detection comes from two aspects—the spontaneous scratch motion from the AD patient population and the exact definition of nocturnal scratch. Feuerstein et al. and Petersen et al.^10,11^ built algorithms from simulated scratches from healthy adults that may not be generalizable to the AD population with spontaneous scratches. Moreau et al.^12^ proposed a bidirectional recurrent neural network classifier to detect scratches among AD patients with improvement from previous studies. However, the reported evaluation metric is based on re-balanced data after over-sampling scratch events, leading to over-estimation of the performance when most of the time contains non-scratch events. Mahadevan et al.^13^ proposed a two-step approach of excluding the period without hand movement and only detecting scratching when hand movement is present. Mahadevan’s method helps to balance the scratch and non-scratch events, but the false negative rate from the movement detection filter is not reported. Therefore, finger scratches can be identified as non-movement and falsely excluded before binary classification. On the other hand, Mahadevan et al. only evaluate scratch during the total sleep opportunity (TSO) window to provide a generalized definition of nocturnal scratch. The TSO window is first defined by van Hees et al.^14^ as the period subjects intend to sleep. It can be detected by actigraphy signal with a heuristic algorithm. In a free-living environment, various scenarios of non-wear or sleep patterns exist. Therefore, a more specific heuristic rule is needed based on the actigraphy signal and the time of the day to capture the “nighttime, intend to sleep” period to define the nocturnal scratch more precisely.

Although many studies explored nocturnal scratch detection from actigraphy devices with accelerometer sensors, few utilized gyroscopes. Gyroscopes measure the device orientation and angular velocity with the additional benefit of motion detection and gesture recognition on top of the accelerometer, especially for low amplitudes motion such as finger scratches^15,16^. In addition, gyroscopes provide a more precise gravity removal from accelerometer measures by getting the device orientation by solving a differential equation for the orientation that depends on the angular velocity^17^. However, the advantage of including a gyroscope sensor in actigraphy devices for nocturnal scratch detection has yet to be fully explored.

To improve the model performance, we advanced both feature extraction and modeling methods. Besides the interpretable features in the time and frequency domain commonly used in literature, we develop additional features with topological data analysis (TDA). TDA, which originated from algebraic topology in pure mathematics, is a rapidly growing field and has proven successful in several scientific areas^18–21^. TDA tools can capture intrinsic shape information, provide effective features or fingerprints, and are robust to noise. Researchers have recently found that using TDA offers different aspects in analyzing time series data^22–25^. We adapt the methods from Chung et al.^22^ to extract topological features. In addition, we ensemble models to boost the performance by deriving extra features from deep learning (DL) models as input to a top-layer LightGBM classifier. Topology-based and DL model-derived features are the top predictors selected by LightGBM.

In this work, we refine the heuristic approach to derive TSO to help define nocturnal scratches more precisely in a free-living environment. We improve the movement detection algorithm to achieve a sensible balance between scratch events prevalence and false negative rate (falsely detect scratch as non-movement). We also demonstrate the benefit of gyroscopes in nocturnal scratch detection by providing more precise gravity removal and additional features. Furthermore, we develop highly predictive topology-based and DL model-derived features to improve model performance. In addition, performance is evaluated on the test set without resampling to reflect the actual performance on imbalanced data, which is most often the case. Finally, digital endpoints measuring the scratch duration and intensities are derived and compared with video annotation ground truth and subjective PROs to demonstrate the objective digital measures’ feasibility, consistency, and reliability.

## Results

### Demographics for study participants

The nocturnal scratch model is built based on 96 nights of data from 20 AD patients. The proportion of male participants is 80%, and the average age is 31 years. The overall disease severity in this study is mild to moderate, with a Severity Scoring of Atopic Dermatitis Index (SCORAD) score mean of 25.8 and an average number of wakings each night of 2. Table 1 includes detailed information on the demographics of study participants.

**Table 1:** Demographics of study participants.

Demographics of 20 participants	Descriptive summary, mean (SD) [range]
Age	30.9 (12.9) [20, 61]
Gender	16M/4F
Race	1 Asian 1 Black or African American 18 White
ADSS – Difficulty to fall asleep (0–4)	1.5 (0.9) [0, 3]
ADSS – Number of awakenings due to itch (0–29)	1.9 (2.3) [0, 15]
ADSS – Difficulty to return to sleep after being woken by itch (0–4)	1.4 (0.8) [0, 3]
SCORAD score (0–103)	25.8 (10.5) [10.2, 54]

*ADSS* atopic dermatitis sleep scale, *SCORAD* severity scoring of atopic dermatitis index.

### Movement detection results

Across all 96 study nights, there are 33,559 s of scratch events from the video annotations with a 0.98% scratch event rate. After applying the movement detection, the scratch event rate increased to 17.8%. The overall performance of the movement detection is shown in Table 2 together with the comparison to the method in Mahadevan et al.^13^. We improved the rate of false detection of scratch as non-movement while greatly increasing the prevalence of scratch compared to the situation without any movement detection filter. The false rate dropped from 36.1% to 10.1% without sacrificing the prevalence too much (prevalence dropped from 20.3 % to 17.8%). There are two possible reasons for those 10.1% of scratches that were falsely detected as non-movement. One is due to low-intensity-scratch such as finger scratches. The scratch pattern varies from person to person, which leads to large variations in subject-level performance, as reported in Supplementary Table S1. The second reason is human error in the video annotation labels. During wrongly annotated periods, the actigraphy signals appear flat without wrist motion but were still annotated as scratches. Given the big challenge in the precision of manual video annotations, it is impossible to avoid this completely. Example plots of these false negative scratch actigraphy signals are shown in Supplementary Fig. S3.

**Table 2:** Performance of movement detection algorithm.

	% scratch wrongly classified as non-movement	Scratch prevalence (%)
No movement detection	0	0.98
Proposed movement detection	10.09	17.84
Movement detection in Mahadevan et al.	36.09	20.28

### Nocturnal scratch classifier

**Fig. 2:**
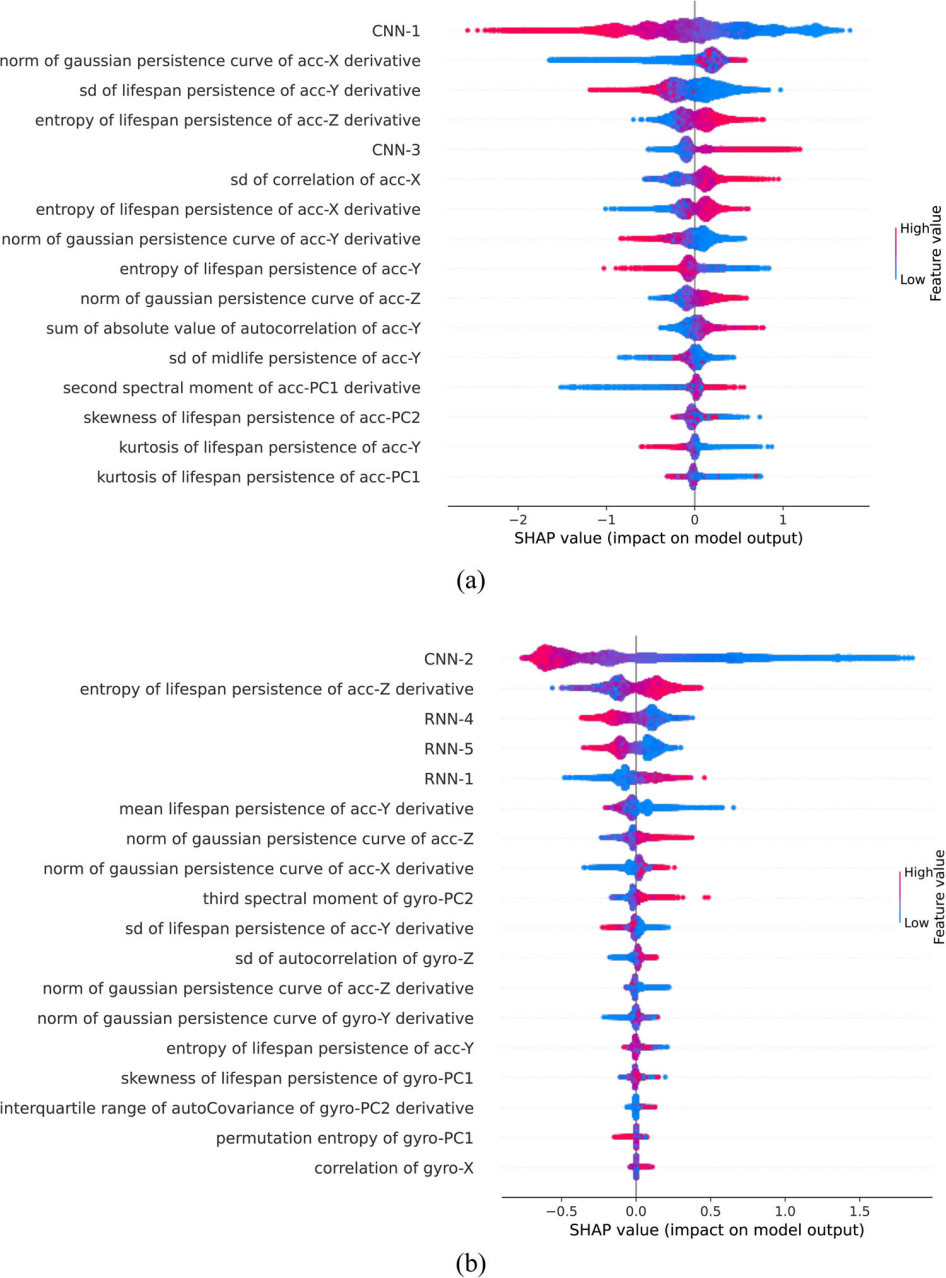
Feature importance. **a** The model with accelerometer data with 16 selected features. **b** The model with both accelerometer and gyroscope data with 18 selected features.

With only 16 features for the accelerometer data model and 18 features for the accelerometer and gyroscope model, the area under the curve (AUC) can achieve above 99% of the AUC of models with all features. In both feature importance plots shown in Fig. 2, topology-based features (persistence statistics and norm of Gaussian persistence curves) are the top selected features for both models. Features derived from DL models are highly predictive as well.

The leave-one-subject-out prediction evaluation is performed within the movement period identified by the movement detection algorithm. Table 3 compares two versions of binary classifiers. The average AUC increased from 0.77 to 0.80 after including the gyroscope signals. The F1 score increased from 0.39 to 0.44. A subject-level evaluation is provided in Supplementary Table S2. The performance difference varies largely among subjects. Subjects with results showing more advantage by adding the gyroscope are likely to have low-intensity motion scratches such as finger scratches. For example, subject 8 benefits from the gyroscope with AUC increased by more than 0.1, and F1 increased by 0.08. Subjects 4 and 6 have AUC increased by more than 0.06 and F1 increased by more than 0.1 and 0.3, respectively. However, subjects 1, 7, and 20 do not differ much, with or without including the gyroscope.

**Table 3:** Leave-one-subject-out evaluation of scratch binary classifier.

	Acc only		Both acc and gyro	
	Mean	SD	Mean	SD
Prevalence	0.18	0.13	0.18	0.13
AUC	0.77	0.05	0.80	0.05
Accuracy	0.76	0.06	0.78	0.05
Recall	0.58	0.17	0.64	0.11
Specificity	0.79	0.09	0.80	0.06
Fl	0.39	0.17	0.44	0.18
PPV	0.35	0.20	0.38	0.21
NPV	0.90	0.09	0.91	0.08

*AUC* area under the curve, *PPV* positive predictive value, *NPV* negative predictive value.

### Digital endpoints validation

**Fig. 3:**
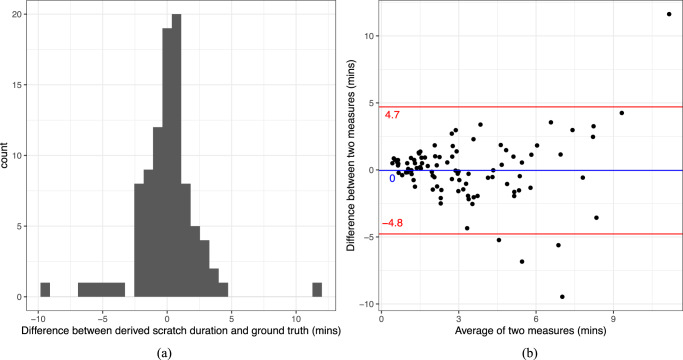
Assessing consistency between ground truth and model-derived endpoints. **a** Histogram of difference between derived scratch duration and ground truth. **b** Bland–Altman plot.

For each subject night, the total scratch duration was derived based on the binary classifier output. The model-derived scratch duration averaged across all 96 study nights is 2.13 s shorter than the ground truth from the video. For most subject-nights, there is no discrepancy between the two measures. From the histogram and Bland–Altman plots shown in Fig. 3, there is no discrepancy or systematic bias in model-derived scratch duration compared to ground truth. The blue line in the Bland–Altman plot is the mean difference between the two measures, and the red lines are the upper and lower limits of the 95% confidence interval for the average difference. There are a few outliers with total scratch duration underestimated by the model compared to video annotation for more than 5 min and one outlier overestimated by more than 10 min.

**Fig. 4:**
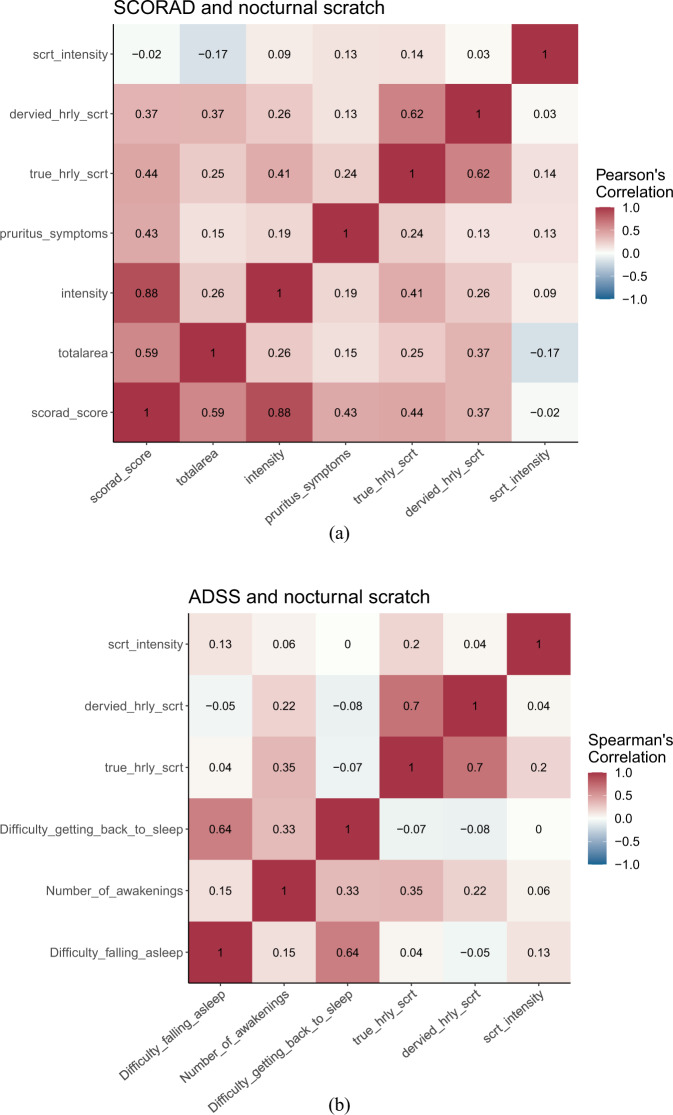
Correlation between patient-reported outcomes and digital endpoints. **a** Correlation between Severity Scoring of Atopic Dermatitis Index (SCORAD) and scratch duration. **b** Correlation between Atopic Dermatitis Sleep Scale (ADSS) and scratch duration.

Overall, there is a weak to moderate correlation between digital endpoints and PROs. Since the patient reports SCORAD only once on their first visit, the correlation shown in Fig. 4 is between SCORAD and subject-level averaged scratch duration. Similarly, the correlation between the SCORAD score and true scratch duration is slightly higher (0.44) than the derived scratch duration (0.37). The correlation between ADSS and digital endpoints is across all 96 nights. The correlation between the number of awakenings and true hourly scratch is 0.35 and slightly higher than the derived scratch duration, 0.22. This result confirms the consistency between digital and PRO measures and also suggests that objective digital endpoints can complement PRO by assessing disease states from different perspectives.

Digital endpoints and ADSS were measured after participants spent each night in the clinic. The intraclass correlation (ICC) shown in Table 4 was calculated based on repeated measures. Both true, from the video annotations, and derived scratch duration have ICC slightly higher than 0.4, indicating weaker consistency or reproducibility compared to the number of awakenings reported by patients.

**Table 4:** Intraclass correlation (ICC).

	ICC
True scratch duration (from video annotations)	0.41
Derived scratch duration (from model)	0.42
Number of awakenings (ADSS)	0.71

## Discussion

The results of this study showed the validity of nocturnal scratch endpoints derived from wrist-worn actigraphy devices in AD patients. Compared to the video annotation ground truth and PROs, nocturnal scratch endpoints accurately capture the disease state. More importantly, nocturnal scratches provide an additional objective assessment of the disease state that subjective measures cannot provide. Given the concern of over-estimation of the scratch detection performance in reality, when the majority of time is non-scratch behaviors, our metric is reported on the dataset without balancing scratch and non-scratch events. Since this study enrolled patients with mild to moderate disease severity, the low prevalence of scratch events makes having a high precision or F1 value more challenging. In a high prevalence event population, it is more likely that a tested positive sample is truly positive than in a low prevalence population. Therefore, we expect higher precision of scratch detection among patients with longer scratch duration. Another path to greatly help to increase the scratch events rate is applying a movement detection filter before the binary scratch classifier. We further improved the movement detection algorithm by lowering the false negative rate compared to the existing method while preserving the purpose of increasing the scratch events rate.

Another innovation of this work is to develop topology-based features from actigraphy signals as predictors for nocturnal scratches. Given the great robustness property of extracting Betti numbers based on the shape of signals, these features are interpretable and highly predictive compared to existing features in time and frequency domains. In the model with the full set of 348 features derived from accelerometer data, 11 out of 16 top predictive features are topology-based. The same idea can also be extended to predicting certain physiological behaviors using wearable device signals.

We also explored the benefit of adding gyroscope data from two aspects—gravity removal and predictive feature extraction. The advantage of gyroscope signals in providing a more precise gravity removal in accelerometer data can also be applied to other actigraphy use cases beyond nocturnal scratch detection. Features extracted from gyroscope signals are highly predictive, given that 7 of 18 selected features are extracted from gyroscope data. Model performance improvement due to adding the gyroscope varies across subjects. This improvement is expected because scratch patterns differ from person to person. In general, including gyroscope signals in nocturnal scratch detection is recommended. Investigating gyroscope benefits in a larger population would be future work. Another big challenge in nocturnal scratch quantification in a free-living environment is to identify the reasonable TSO window, especially when patients may fail to wear devices on both wrists all the time. We extended the existing approach of identifying TSO for each hand separately to getting a combined TSO from both hands when valid data are available. Otherwise, if a subject only wears a device on one wrist, TSO and digital endpoints are only derived from that hand. In addition, we leveraged movement detection and temperature information to identify the non-wear period and, thus, better identify the TSO in a free-living setting.

Finally, we examined the validity of derived digital endpoints analytically and clinically by comparing them with the true scratch duration from video annotations and PROs. The model-derived scratch duration is consistent with ground truth without any systematic bias. The weak to moderate correlation between digital endpoints and PROs indicates that the digital endpoints are consistent with existing PROs and can assess disease states from different aspects than PROs. The weaker ICC of digital endpoints compared to PROs is due to the finer granularity that digital endpoints measure from night to night. Therefore, collecting device data and averaging them across one to two weeks from each patient is recommended for studies using actigraphy to assess disease state.

Given that nocturnal scratch is detected based on wrist motions in this work, there is a limitation in capturing other formats of scratching. Some examples include non-hand scratches and rubbing part of the body on a pillow, sheets, or other bedding. To fully consider non-hand scratches, we need to leverage other sensor technologies. Similar to many existing studies, the sample size is also a limitation. To further improve the algorithm’s robustness and generalizability, refining the algorithm with more subjects and across different disease severity groups will be future work. In addition, the ability to assess treatment efficacy through digital endpoints is not yet fully explored. A future study is required to collect actigraphy data from patients while taking treatment with known efficacy.

## Methods

### Study design and data collection

There were 23 AD patients enrolled with up to five nights of data collected from each participant, and three subjects were excluded due to device malfunction and software issues. We have 96 nights of data from 20 subjects to train the model. During each study night, participants slept at the clinical research unit (CRU) on a mattress with an EMFIT bed sensor, wore an AX6 actigraphy device on each wrist, and had an infrared camera videotape throughout the night. Triaxial accelerometer and triaxial gyroscope signal data were collected from AX6 to capture the wrist motion of both hands. Two independent reviewers annotate videos with an arbitrator to reconcile the discrepancy. The data source and description are shown in Table 5. WIRB-Copernicus Group Institutional Review Boards approved this study. All enrolled participants provided written informed consent approved by the ethical review board governing the CRU.

**Table 5:** Description of data source.

Data source	Data type	Data description
Actigraphy (AX6)	Device signal (6-axis)	Accelerometer and gyroscope sensor from wrist-worn device running at 50 Hz sampling frequency
Bed sensor (EMFIT)	Device signal	Pressure-sensitive sensor under mattress running at 50 Hz, sleep stages can be derived for each second. Total sleep opportunity window can be determined with EMFIT
Infrared camera	Video	Video (24 fps) records patients’ behaviors
Video annotation	Events with timestamps and label	Video recordings are annotated by two independent reviewers and reconciled by an arbitrator based on the video to the level of milliseconds. Each annotation includes event label (“scratch,” “non-scratch movement,” “in-bed still,” “out-of-bed”), start and end timestamps, hand information. This is the ground truth of nocturnal scratch model
ADSS	Patient-reported outcome	Atopic Dermatitis Sleep Scale is a 3-item questionnaire reported by patients daily. Item 1 and 3 are assessing “difficulty of falling asleep due to itch" and “difficulty of getting back asleep due to itch" with a 5-point Likert-type scale ranging from 0 “not at all" to 4 “very difficult." Item 2 records the number of times patients woke up each night and ranges from 0 to 29 times
SCORAD	Patient-reported outcome	Severity Scoring of Atopic Dermatitis Index (SCORAD) is reported by patients once at the screening visit. SCORAD ranges between 0 and 103 to assess the severity of AD from aspects including body area affected, condition intensity, itch and insomnia

### Algorithm overview

**Fig. 5:**
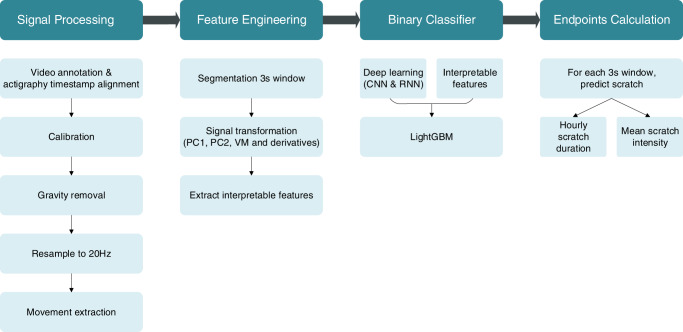
Overview of proceess from signal processing to endpoints calculation. Overview of signal processing, feature engineering, modeling, and digital endpoints calculation.

Given the scope of nocturnal scratch detection, the first step is identifying the TSO window. The TSO window is defined as the period of nighttime in which participants are ready for sleep or trying to sleep. In this study, participants were instructed to wear the actigraphy devices only during the nighttime after arrival at the CRU. After participants woke up and left the CRU in the morning, devices were placed on the table (still turned on). In this case, TSO is identified by the EMFIT bed sensor. The moment when participants sit or lay on the mattress each study night is the start of the TSO window, and the moment when participants leave their bed in the morning is the end of the TSO window. We also developed a rule-based approach using accelerometer and temperature data to obtain TSO for the case without bed sensors and participants wearing devices for 24 h^13,14^. After getting the TSO window, we followed the steps shown in Fig. 5 to process signals, extract features, build models, and derive endpoints. Given that the AX6 device collects both accelerometer and gyroscope data, in signal processing and modeling steps, we built two versions for comparison—version one involves accelerometer data only, and version two uses both accelerometer and gyroscope data. Detailed descriptions for each step are discussed in the following subsections.

### Signal processing

**Fig. 6:**
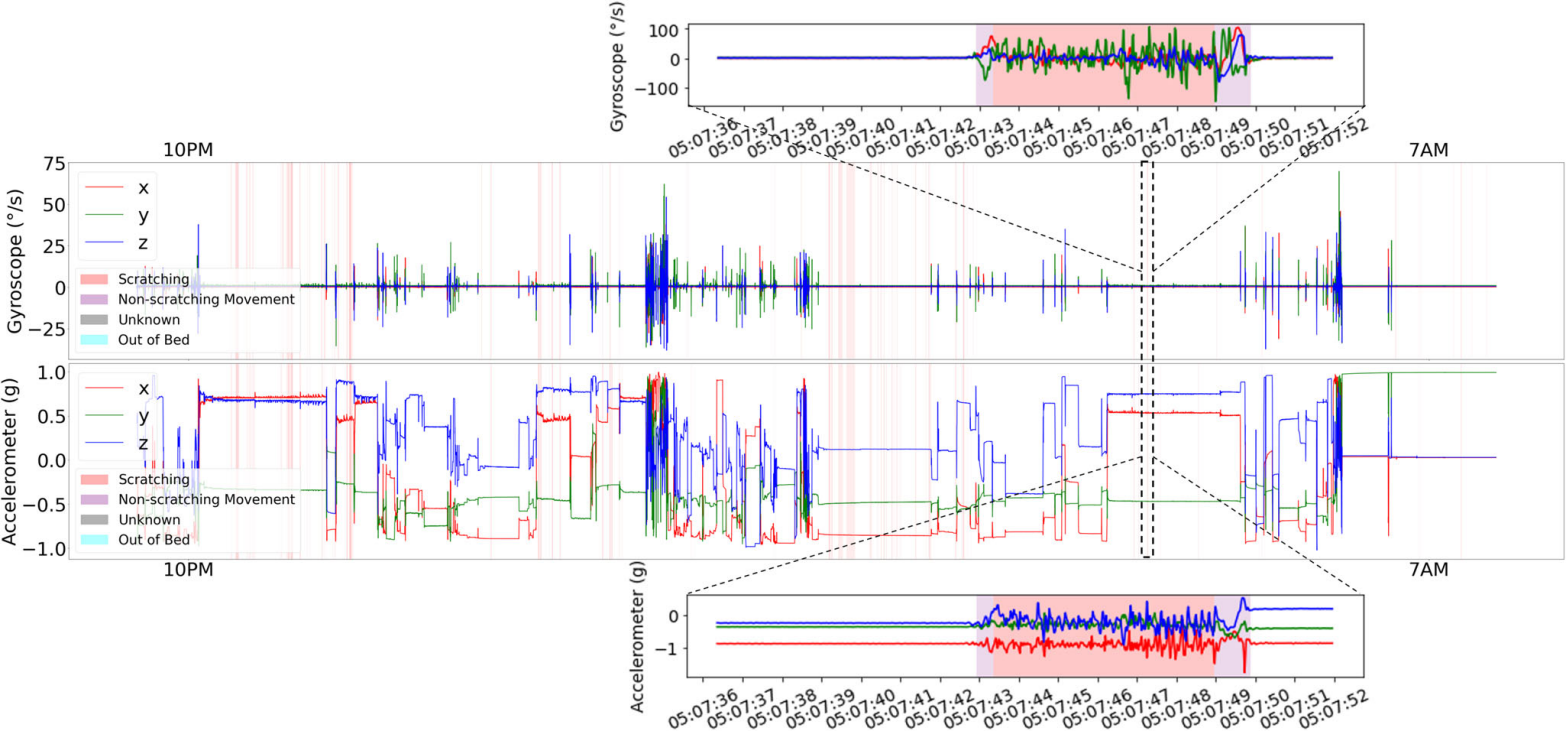
Overlaid actigraphy data and video annotations. Plots detailing one subject night of actigraphy data overlaid with video annotation after timestamp alignment. Note: Slight differences between the video annotation of the accelerometer and gyroscope plots are due to rendering.

To ensure the model was trained with correctly annotated data, the first step was to align the timestamps between the actigraphy device and video annotation for each study night. Factors such as initial actigraphy device configuration and drift from the internal real-time clock (RTC) can impact the alignment. The initial time configuration of the AX6 device is synced to the time of the connected computer to the nearest second. In this case, the connected computer may have been set to a different time zone, which caused hours of alignment discrepancies. Under normal operating conditions, the RTC drift for AX6 is specified to be up to ±4.32 s per day. To visually align for initial device configuration and RTC drift, we plot accelerometer and gyroscope signals along with the annotation. Then, we visually measured the amount of alignment correction needed based on the movement patterns in the sensor signals for each subject- night to the nearest second. The amount of RTC drift during a night is typically less than ±1 s, so we did not correct for any slight variations of RTC drift within each night. We used the visual measurements to shift the AX6 actigraphy data to align with the video annotations. Figure 6 shows a sample of the actigraphy and video annotation data after alignment.

The raw accelerometer signal was calibrated to local gravity to account for the device-level noise^26^. Since we are interested in patient movement, not, e.g., hand orientation in a global reference frame, we need to remove gravity from the measured acceleration. To this end, we employed two distinct approaches, depending on whether the downstream model leverages gyroscopic information. Many clinicians may opt to forgo gyroscope utilization due to the additional energy burden placed on the device that requires more frequent device charging and potential issues with patient compliance. Unfortunately, it is impossible to remove gravity exactly with only accelerometer data. We followed the standard approximation for this version and used a high-pass first-order Butterworth filter with a cutoff frequency of 0.25 Hz. When we also have gyroscopic information, it is possible to remove gravity exactly (up to device noise and numerical drift). Here we used an in-house approach that solves a quaternion ODE that leverages stationary regions with sensor fusion to mitigate drift, as described in Supplementary Information Section 1.

**Fig. 7:**
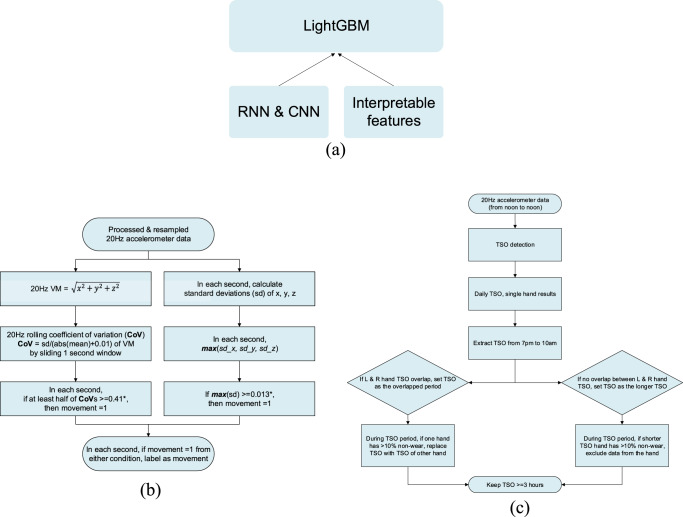
Detailed diagrams of pipeline. **a** Ensemble deep learning model with LightGBM. **b** Movement detection algorithm; * 0.41 and 0.013 were chosen based on quantiles from the entire dataset. **c** Steps of getting combined TSO from both hands.

Similar to the approach taken in Mahadevan et al., our model aims to detect scratches generated by hand or wrist motion. Thus, a threshold-based filter was applied to exclude the period without hand movement. The model only classifies scratch and non-scratch within the period with any hand or wrist-related motion present. As scratch only happens with hand motions, this reduces non-scratch time and helps to increase scratch labels in train and test sets, especially in the mild disease state population. We compared two versions of the movement algorithm with and without gyroscope data and observed a small difference in performance. The current movement detection algorithm is based on accelerometer data only to simplify the approach and avoid extra parameters. Figure 7 shows the process of movement extraction. After resampling raw data to 20 Hz sampling frequency, we computed vector magnitude (VM) $\sqrt{{X}^{2}+{Y}^{2}+{Z}^{2}}$ within each 1-s window. Both low pass filter (6th order with 3 Hz cutoff) and high-pass filter (1st order with 0.25 Hz cutoff) were applied to remove noise and constant in VM, respectively. The movement period was detected based on two threshold quantities. The first quantity is the rolling coefficient of variation (CoV) computed by sliding a 1-s window. The second quantity is the maximum standard deviations (SD) among the *X*, *Y*, and *Z* axes within each 1-s window. For each second, if more than half of the CoV is greater than 0.41 and the maximum SD is greater than 0.013, then we marked this second as hand movement. The choice of thresholds reflects a trade-off between how much true scratch is falsely excluded and the percent of scratch in train and test sets. Here 0.41 and 0.013 were picked as the 8% quantiles from the entire dataset to achieve a good trade-off.

### Feature engineering

The extracted movement period was further segmented into 3-s windows with 1.5-s overlap to increase the sample size. The literature suggests that 3-s windows achieve a good trade-off between temporal resolution and detection performance^10,11,13^. The binary label for each 3-s window was generated based on video annotation. A window with more than 1-s scratch present was labeled as a scratch. Edge cases (27.6 %) with a 3-s window covering both movement and non-movement periods are also included, and video annotations determine their labels.

Accelerometer and gyroscope sensor signals were transformed by computing VM, first and second principal components (PC1, PC2) to make the signal orientation invariant. This transformation eliminates the dependence on wrist orientation by effectively changing the signal basis into the two-dimensional plane that scratch occurs^13,16^. For each sensor, we have 12 channels of signals (*X*, *Y*, *Z*, PC1, PC2, VM, and their corresponding derivatives) as input to extract features.

There are two types of features extracted from the transformed signals. The first type of feature is interpretable with meanings in the time and frequency domains to reflect the signal’s range, periodicity, smoothness, and other properties. In addition to those commonly used features in literature^10,11,13,16^, we developed additional features with TDA. TDA is a rapidly growing field and has proven successful across various scientific disciplines^21,27^. Recently, researchers have also found that using TDA tools offer different aspects in analyzing time series data^22–25,28^. In this work, we consider 11 TDA-based features, and 10 of them are called persistence statistics^22^, and they are summary statistics of two sets of numbers lifespan persistence and midlife persistence. The last feature is the norm of the Gaussian persistence curve^29^. Details about these 11 features can be found in Supplementary Information Section 2. We use the GUDHI package for computing persistence diagrams^30^. The second type of feature is non-interpretable and derived from DL models. We derived ten features by taking the penultimate layers from the convolutional neural network (CNN) and recurrent neural network (RNN) models. In total, we have 348 features for the model with only accelerometer data, where 338 are interpretable features and 10 are layers from CNN and RNN models. The model with accelerometer and gyroscope data has 686 features, 676 are interpretable, and 10 are layers from CNN and RNN models. Feature selection has been performed with recursive feature elimination. For the accelerometer data model, 16 features are selected. For the model with both accelerometer and gyroscope data, 18 features are selected. This decision is based on the elbow plots shown in Supplementary Fig. S2.

### Nocturnal scratch detection model

A binary classifier is trained to detect scratch events. We employed a hierarchical ensembling approach with LightGBM as the top layer. Lower levels consist of physics-based, topology-based, and DL model-derived features (CNN/RNN), as shown in Fig. 7a. This ensembling allows us to effectively leverage the inductive biases offered by a variety of different approaches in order to boost the overall performance. For the ML-based features, we used modest architectures for our CNN and RNN. The CNN consists of two layers of convolution, batch normalization, and max pooling, followed by a 3-layer MLP with output dimensions (16,5,1). The RNN consists of a bidirectional LSTM layer again, followed by a 3-layer MLP with layer output dimensions (16,5,1). In each case, we trained the model to classify scratch, remove the final layer from the trained network, and use the penultimate layer output as a feature. We chose a conservative hidden dimension for the penultimate layer to obtain five distinct features from each model.

Given that the future deployment of this model is on new patients, we evaluate the leave-one-subject-out performance to mimic reality. For each fold, we hold out data from one subject. For the rest subjects, we randomly split data to train and validation sets with an 8–2 ratio. The validation set is used to evaluate early stop criteria to prevent model overfitting. Hyperparameters, including the number of leaves and the tree’s maximum depth, were tuned with the train set. Since participants in this study had mild to moderate disease states, the scratch events prevalence is low. To address this, “scale_pos_weight" has been included as a hyperparameter to over-weight scratch samples to balance the train set. However, we kept the original prevalence in the test (hold-out) set for evaluation to reflect the actual performance in reality, especially for mild to moderate AD patients, where scratch events are rare. Feature selection has been performed with recursive feature elimination within each fold to reduce dimension. To evaluate the additional benefit of the gyroscope sensor, we fit the model with two sets of features for comparison. The first set includes features processed and extracted only from accelerometer data. The second set includes features extracted and processed with accelerometer and gyroscope data.

### Model deployment, digital endpoints extraction and analysis

**Fig. 8:**
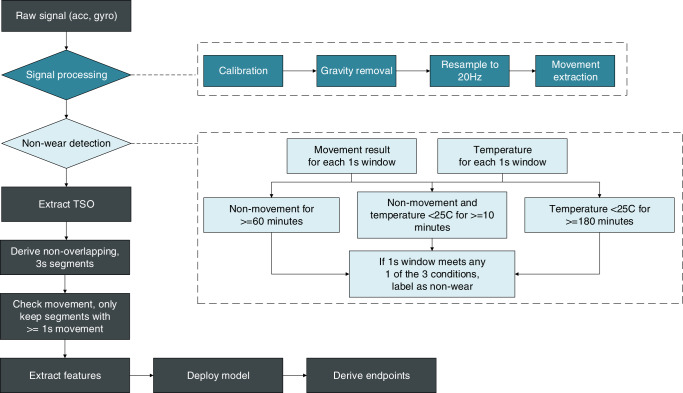
Model deployment. Flowchart of model deployment.

This subsection describes the steps to deploy the model and derive nocturnal scratch endpoints using the proposed approach. After processing the raw signal with calibration, gravity removal, and resampling to 20 Hz, the movement detection filter is applied to exclude all non-movement periods. Then, the time of non-wear, or when the actigraphy device is not worn on the wrist, is identified based on temperature and non-movement, as seen in Fig. 8. For example, if a non-movement period has a temperature lower than 25 Celsius for more than 10 min, it is considered a non-wear period. Next, the TSO periods are retrieved. For studies without information on TSO start and end time, the TSO can be identified by the heuristic algorithm proposed by van Hees et al.^14^ together with rules in Fig. 7c to obtain a combined TSO from left and right hands that cover nighttime only. After excluding all non-wear time within the TSO window, the time series data are further segmented into 3-s, non-overlapping windows to apply the movement detection algorithm. All 3-s windows with at least 1-s identified as a movement have features extracted and passed as input to the binary classifier to detect scratch.

For each subject night, two endpoints are extracted to measure scratch duration and intensity based on the binary classifier output. Information from both hands is pooled together. Hourly scratch duration is computed as the average scratch duration, in seconds, within each hour within the TSO window. Averaged scratch intensity is the mean dominant frequency value, in Hz, of all scratch windows.

Analyses are applied to evaluate the digital endpoints’ accuracy, reliability, and consistency. First, the endpoints derived from models are compared against ground truth endpoints from the video annotations with correlation. Second, the association between digital endpoints and PROs (SCORAD and ADSS) is assessed. Finally, the ICC coefficient is used to assess test-retest reliability to show whether digital endpoints are reproducible for the same patient across time points with a similar disease condition.

### Platform and pipeline

Digital endpoints, such as the objective and quantifiable measure of nocturnal scratch duration and density, offer unique insights into AD patients’ disease states and life quality. However, the opportunity comes with its unique challenges. Data we collect in this study (and many others) includes sensor signals from actigraphy, PROs from hand-held devices, and video annotation ground truth information for algorithm development and model building. Handling these data is a big data problem. For example, with a sampling frequency of 50 Hz, over 4 million 3-axial data points are collected from both the accelerometer and gyroscope sensors for a single day to understand a patient’s daily activities. Developing various measures requires an iterative process and repeated trial and error cycles to develop, validate, and confirm our hypothesis. Discovering accurate and meaningful measures also requires data aggregation across different levels, which can often be tedious and error-prone. One emerging capability in the industry is a fit-for-purpose ecosystem to collect, visualize, analyze, and report digital datasets efficiently and effectively across the typical data life-cycle to develop desired digital endpoints.

**Fig. 9:**
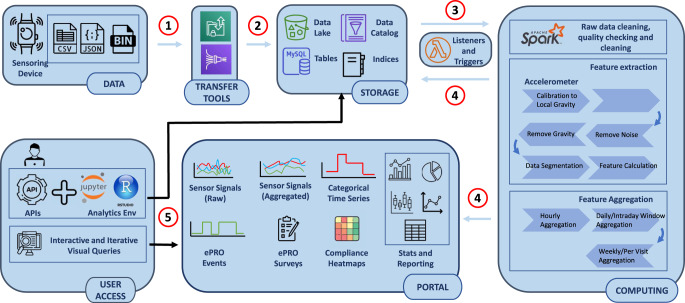
A typical data flow in data platform. ①–⑤ indicate the data flow sequence order: ① digital data ingestion, ② digital data landing in DDR repository and getting structured, ③ newly arriving digital data triggering micro-services for further processing and transformation, ④ data reporting and visualization being updated with newly arriving digital data and derived measures, and ⑤ digital data delivered through APIs.

Toward this goal, we developed a home-grown digital data platform at Eli Lilly & Company. The typical data flow enabled by this platform is shown in Fig. 9. Sensor signals and PRO data arrive at our platform’s storage layer through high-performance transfer tools and services. Once data lands, various processing pipelines are triggered, either in parallel (when there is no dependency) or in a particular order (i.e., chained pipelines when the sequence matters). One example is a spark pipeline for raw data cleaning and quality checking, followed by concurrent feature extraction pipelines, each of which handles a specific sensor data type, e.g., our primarily used actigraphy data processing pipeline that involves the following modules: (1) calibration to local gravity; (2) resampling; (3) gravity removal; (4) noise removal; (5) data segmentation; and (6) features calculation. Once data from all channels (e.g., accelerometer, gyroscope) are ready, a data aggregation pipeline launches to perform aggregation at different levels, from hourly, daily, weekly, or per visit in the study.

**Fig. 10:**
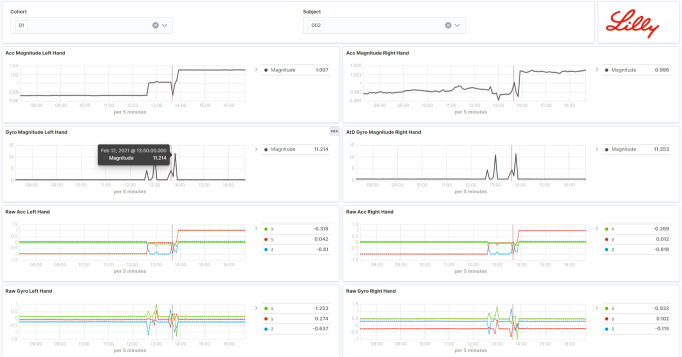
An example of data visualization. Raw accelerometer and gyroscope sensor signals and their derived magnitudes of each participant’s hand are displayed and can be interactively explored.

Data artifacts from running data pipelines are pushed automatically into the storage layer and the data portal (see, e.g., Fig. 10 for a complete sensor datasets collected from a participant in this study), making them immediately accessible through graphical web interfaces or programmatically via APIs for digital measure development and validation.

### Reporting summary

Further information on research design is available in the Nature Research Reporting Summary linked to this article.

## Supplementary information

Supplementary Information

Reporting Summary

## Data Availability

Upon request, and subject to review, Eli Lilly and Company will provide the aggregated and raw data that support the findings of this study.

## Code availability

Analysis of datasets was performed with Python version 3.6+ and R version 4.2.2, and code will be shared upon request.
