# Supplementary material for: Assessing nocturnal scratch with actigraphy in atopic dermatitis patients
Source: NPJ Digit Med. 2023 Apr 26;6:72. doi: 10.1038/s41746-023-00821-y (PMC10133290; doi:10.1038/s41746-023-00821-y)
Supplement: Supplementary file 1 — Supplementary Information [file 41746_2023_821_MOESM1_ESM.pdf]

Supplementary Information for
Assessing Nocturnal Scratch with Actigraphy in Atopic Dermatitis Patients

Contents

1 Gravity Removal **2**

2 Features Extraction with Topological Data Analysis **4**

3 Feature Selection **6**

4 Movement Algorithm and Scratch Detection Model Results **7**

5 Distributions of ADSS and SCORAD **11**

1 Gravity Removal

Quaternion Approach

Accelerometers measure dynamics, *e.g.*, acceleration, and angular velocity, with respect to the device orientation. For example, measurements are recorded with respect to the local (body) reference frame. So, as the device is rotated in the global reference frame, the acceleration due to gravity picked up by the accelerometer is generally not static with respect to the local frame. Thus if we want to remove gravity, we effectively need to know the device's orientation. Once this is known, we can trivially remove gravity by projecting it out. The problem is then reduced to determining the device orientation. This may be done by solving a differential equation for the orientation depending on the angular velocity (the instantaneous change in orientation measured by the gyroscope in the local frame). Our strategy will thus be as follows: (1) identify stationary and non-stationary regions of signal through e.g., standard deviation thresholds, (2) estimate the device orientation in the stationary regions, (4) use the estimated orientation from stationary regions as an initial value for the ODE used to find the orientation in the adjacent non-stationary regions, and (5) project out gravity.

We formulate our approach in terms of quaternions due to their nice theoretical properties. Quaternions constitute a representation of the 3D rotation group, SO(3), and may be used to encode these rotations more efficiently and stably than rotation matrices with Euler angles. Ostensibly quaternions are 4D vectors with a scalar and vector component whose components form a 4D associated normed division algebra over $\mathbb{R}$. A rotation of angle θ about the unit vector $\mathbf{n}$ may be written as a quaternion:

$$R = e^{\frac{\theta}{2}(n_x\mathbf{i}+n_y\mathbf{j}+n_z\mathbf{k})} \tag{1}$$

If we consider vectors in $\mathbb{R}^3$ to be quaternions with zero scalar component, the rotation of a vector $\mathbf{v}$ by a quaternion $\mathbf{R}$ is given by

$$\mathbf{v}' = \mathbf{R}\mathbf{v}\mathbf{R}^{-1} \tag{2}$$

The literature [1, 12] derives the following ODE for quaternion evolution using the angular velocity in the local reference frame:

$$\frac{d\mathbf{R}}{dt} = \frac{1}{2}\mathbf{R}\omega \tag{3}$$

It is important to realize that because quaternions do not commute, a closed-form solution via e.g., exponentiation is not readily possible. Then if $\mathbf{g}(t)$ is the gravity vector in the local frame, $\mathbf{g}(t) = \mathbf{R}(t)\mathbf{g}(0)\mathbf{R}^{-1}(t)$ where the dynamics of $\mathbf{R}(t)$ are governed by Supplementary Equation 3 and $\mathbf{g}(0)$ is the initial orientation obtained by an adjacent stationary region where we have high confidence in the orientation (we believe the only contribution to acceleration is from gravity itself).

Mitigating Drift Error

While the above theory is, in principle, correct, it is plagued by errors from device precision and finite timestep size in solving the ODE (drift). If these are not addressed, the deviation of the estimated device orientation from the true orientation can be substantial, especially far from the stationary regions. So, to effectively remove gravity, we must add some error corrections. One easy way to do this is to estimate the gravity vector's orientation with rough estimates of error and fuse it with the predicted dynamic value from the ODE with the estimated drift error. Getting good error estimates can be tricky, but a heuristic approach appears sufficient. We get estimates of the gravity orientation by heavily filtering the acceleration component-wise with a low-pass Butterworth filter (f_c = 0.4 Hz) and then normalizing at each timestep. We approximate the uncertainty of the estimate as $u(t) = (||\mathbf{a}|| - 1)^2 + 0.1||\omega||^2$ where ω has units rad/s and acceleration, $\mathbf{a}$, has units m/s^2. Note that this is only a qualitative estimate with the property that it is small for regions near stationarity and large otherwise. The overall scale relative to the estimated drift error controls the Kalman gain of our fusion /textiti.e., the extent to which $\mathbf{R}(t)$ consists of a contribution from the provided estimate or the ODE. In order to further emphasize the role of stationary regions, we

also pass the uncertainty, $u(t)$, through a smoothed Heaviside filter: $u \leftarrow \sigma\Big(100(u(t) - .05)\Big) * u(t)$ where $\sigma(x) = \frac{1}{1+e^{-x}}$.

Let $\mathbf{R}_{est}(t)$ and $\mathbf{R}(t)$ be the quaternions that rotate $\mathbf{g}_{est}(0)$ to $\mathbf{g}_{est}(t)$ and $\mathbf{g}_{est}(0)$ to $\mathbf{g}(t)$ respectively where $\mathbf{g}_{est}(t)$ is the estimated gravity at each timestep, t, and $\mathbf{g}(t)$ is the dynamic gravity vector solved from our ODE and fused with the estimate. We define $e(t)$ as our propagated error with $e(0) = u(0)$ and e_{ODE} to be the estimated error from the discretization of the ODE, then $e(t+1) = \sqrt{e(t)^2 + e_{ODE}}$. The Kalman gain [17] is $g = \frac{e(t)^2}{e(t)^2 + u(t)^2}$. Quaternion fusion is performed by $\mathbf{R}(t) = \mathbf{R}(t-1) + g * \Big[\mathbf{R}(t-1) * \mathbf{R}_{mod}(t-1) - \mathbf{R}(t-1)\Big]$ where $\mathbf{R}_{mod}$ is the quaternion from the ODE that would update the previous quaternion with the smallest rotation. The propagated error must then be updated to account for the effect of fusion via $e(t) \leftarrow e(t)\sqrt{1-g}$. This gives the desired prescription for solving the ODE while leveraging regions near stationarity to mitigate drift.

2 Features Extraction with Topological Data Analysis

Topological Data Analysis (TDA) is a rising field in the intersection of pure mathematics, statistics, and machine learning [4, 6, 10, 11, 23, 24]. It concerns the "shape" of data in the form of Betti numbers, a classic subject in algebraic topology. Persistence diagrams, one of the main tools from TDA, have been proven effective and successful in many scientific disciplines (see survey article [16] for a list of applications). Recently, researchers have also found that TDA offers different aspects to analyze the time series data [7, 13, 14, 18–20, 22]. In this work, we consider the sublevel set persistence diagram for a time series and extract features from it. Mathematical details about sub-level set filtration and persistence diagram can be found, e.g., in [5]. This Supplementary document illustrates these mathematical concepts by an example and describes the TDA features we used in the main document.

We first describe the definition of the sublevel set. Given a function $f : \mathbb{R} \to \mathbb{R}$ and a threshold t, the sublevel set of f at t is defined as

$$f_t := \{(x, f(x)) \mid f(x) \leq t\}.$$

Formally, a given threshold t can be thought of as a horizontal line ($y = t$) in the plane and the sublevel set at t is the portion of f below the horizontal line. For example, Supplementary Figure S1(b)-(h) show sublevel sets of f at threshold values 2, 6, 7, 9, 12, 13, and 18, respectively, and they are depicted in blue color. The foundation of TDA tools concerns the Betti numbers [REF] of sets. Betti numbers are the number of disjoint connected components or isolated regions in a sublevel set. For instance, Supplementary Figure S1(e) shows the sublevel set f_9 depicted in blue color, and we observe that there are four isolated regions, and thus, the Betti number of f_9 is 4. Similarly, Betti numbers of f_2, f_6, f_7, f_9, f_{12}, f_{13}, and f_{18} are 1, 2, 3, 4, 3, 2, 1.

Second, we describe the relation among sublevel sets. Take the function f depicted in Supplementary Figure S1(a) as an example. As shown in Supplementary Figure S1(b), $f_2 = \{(4, 2)\}$ is a set of single point since the threshold value 2 is the global minimum of f. As shown in Supplementary Figure S1(c)-(h), as the threshold value t increases, we observe that the sublevel set increases, too, *i.e.*, more of f will be included as t becomes larger. It can be shown that

$$f_{t_1} \subseteq f_{t_2}, \text{ for any } t_1 \leq t_2,$$

and this relation is called the sublevel set filtration. Tools in TDA concern tracking changes of Betti numbers in a filtration. Persistence diagrams of the sublevel set filtration are the media to store such information. We now describe the sublevel set persistence diagram for a function by the following example. As we have seen Supplementary Figure S1(b)-(h), sublevel sets change over threshold values t. The persistence diagram stores information about those changes in the sublevel sets in the form of birth and death values. We illustrate this idea by the example in Supplementary Figure S1. The process starts from the smallest value of f (Supplementary Figure S1(b)) to the largest value of f Supplementary Figure S1(h)). During the process, we focus on tracking how the sublevel set appears (is born) or disappears (dies). In Supplementary Figure S1(b), f_2 contains a single point depicted as blue color and labeled as Roman "I". In Supplementary Figure S1(c), we see that the region "I" grows and that a new region is born "II". In Supplementary Figure S1(d), we see that both regions "I" and "II" grow, and we also observe that a new region is born "III". In Supplementary Figure S1(e), we see that regions "I", "II", and "III" grow, and we also observe that a new region is born "IV". In Supplementary Figure S1(f), we see that region "I" and "IV" merge, and since "IV" was born later than "I", we say that "IV" dies at threshold value 12. In Supplementary Figure S1(g), we see that region "II" and "III" merge and since "III" was born later than "II", we say that "III" dies at threshold value 13. Lastly, in Supplementary Figure S1(h), we see that region "I" and "II" merge, and since "II" was born later than "I", we say that "II" dies at threshold value 18. We observe that the region "I" actually never dies. By the convention in the TDA field [10], we say that the region "I" has the death value ∞. Therefore, the persistence diagram is $D = \{(9, 12), (7, 13), (6, 18), (2, \infty)\}$. Algorithms for computing persistence diagrams are well-studied. We use GUDHI [21] to compute persistence diagrams in this work.

We have seen that a persistence diagram, denoted by $D = \{(b, d)\}$, is a multi-set that contains birth (b) and death (d) coordinates for each topological feature in the sub-level set filtration. The next step is to

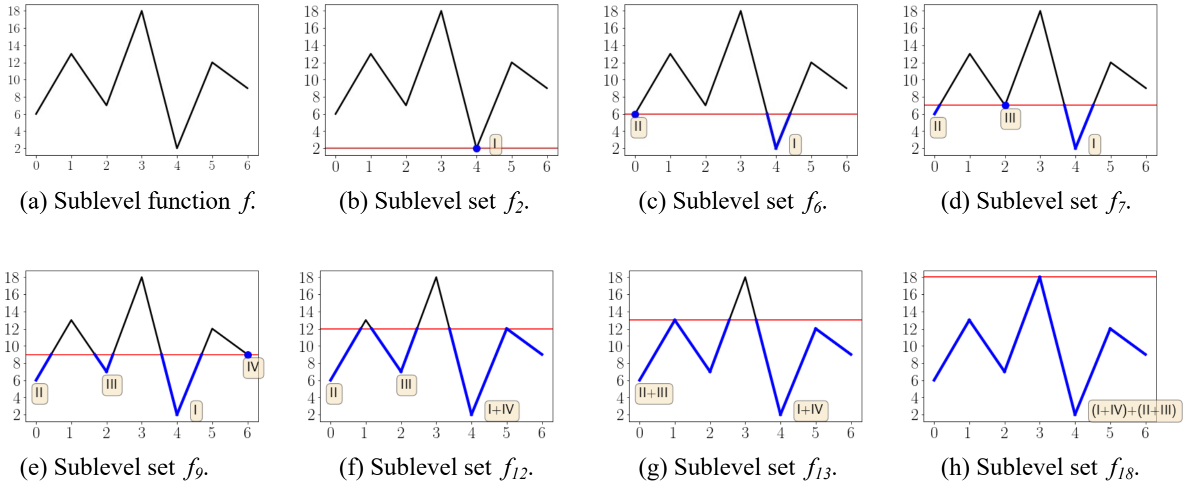

(a) Sublevel function f. (b) Sublevel set f_2. (c) Sublevel set f_6. (d) Sublevel set f_7.

(e) Sublevel set f_9. (f) Sublevel set f_{12}. (g) Sublevel set f_{13}. (h) Sublevel set f_{18}.

Supplementary Figure S1: Demonstration of sublevel set persistence diagram. D = (9, 12), (7, 13), (6, 18), (2,). In particular, "I" corresponds to the pair (2,); "II" corresponds to the pair (6, 18); "III" corresponds to the pair (7, 13); "IV" corresponds to the pair (9, 12).

utilize persistence diagrams to perform machine learning algorithms. However, directly applying machine learning algorithms on PDs is a challenging task due to lacking Hilbert space structure [3,15]. Summarizing or vectorizing the persistence diagram is a common approach to mitigate the difficulty and is currently an active research area in the field (see *e.g.*, [2, 9]). In this work, we consider persistence statistics [7] and Gaussian persistence curve [8]. For persistence statistics, we consider two sets of numbers: lifespan persistence $L = \{(d-b) \mid (b,d) \in D\}$ and midlife persistence $M = \{(d+b)/2 \mid (b,d) \in D\}$. For each set of numbers M and L, we compute five basic summary statistics: the sample mean, standard deviation, skewness, kurtosis, and entropy. In addition to persistence statistics, we consider the norm of Gaussian persistence curves [8], which is defined below:

$$\|G_D\|_1 = \sum_{(b,d)\in D} \left[(d-b)\Phi(\frac{d-b}{\sqrt{2}\sigma}) + \sqrt{2}\sigma\phi(\frac{d-b}{\sqrt{2}\sigma}) \right], \tag{4}$$

where $\phi(x)$ is the probability density function of standard normal distribution and $\Phi(x)$ is the cumulative density function of standard normal distribution. In summary, for each time series, the TDA-based features are 11 dimensional, and they are mean(L), std(L), sk(L), ku(L), epy(L), mean(M), std(M), sk(M), ku(M), epy(M) and Supplementary Equation 4. A sample python implementation can be found in Listing 1.

Take Supplementary Figure S1 as an example. In practice, we treat ∞ as the largest value in the time series. Thus, in this example, "∞" will be replaced by 18, so $D = \{(9,12),(7,13),(6,18),(2,18)\}$. By direct calculation, we obtain the lifespan persistence $L = \{3,6,12,16\}$ and midlife persistence $M = \{10.5, 10, 12, 10\}$. We then compute the mean, standard deviation, skewness, kurtosis, and entropy for M and L, respectively to obtain $[10.625,\ 0.819,\ -0.851, 0.957,\ 1.38,\ 9.25,\ 5.068,\ -1.571,\ 0.095,\ 1.226]$, and by Supplementary Equation 4 to get $[(12-9)\Phi(\frac{12-9}{\sqrt{2}}) + \sqrt{2}\phi(\frac{12-9}{\sqrt{2}})] + [(13-7)\Phi(\frac{13-7}{\sqrt{2}}) + \sqrt{2}\phi(\frac{13-7}{\sqrt{2}})] + [(18-6)\Phi(\frac{18-6}{\sqrt{2}}) + \sqrt{2}\phi(\frac{18-6}{\sqrt{2}})] + [(18-2)\Phi(\frac{18-2}{\sqrt{2}}) + \sqrt{2}\phi(\frac{18-2}{\sqrt{2}})]$ when choosing $\sigma = 1$.

```python
import numpy as np
from scipy.stats import norm
import scipy
import gudhi

def sublevel_set_persistence_diagram(ts):
    """
    Compute sublevel set persistence diagram for a given time series.
    Args:
        ts: input time series in an n-by-1 array.
    Returns:
        persistence diagram in a n-by-2 array.
    """
    Complex = gudhi.CubicalComplex(dimensions=[len(ts),1], top_dimensional_cells=ts)
    Complex.persistence()
    D0=Complex.persistence_intervals_in_dimension(0)
    return D0

def get_GPC_norm1(D, s):
    """
    Compute 1-norm of Gaussian persistence curve for a given persistence diagram.
    Args:
        D: persistence diagram in an n-by-2 array.
        s: parameter for the persistence curve.
    Returns:
        1-norm of Gaussian persistence curve, a scalar.
    """
    b = D[:,0]
    d = D[:,1]
    gpc_norm1 = np.sum( (d-b) * norm.cdf( (d-b)/np.sqrt(2*s*s),0,1 )
                        + np.sqrt(2*s*s)*norm.pdf((d-b)/np.sqrt(2*s*s),0,1) )
    return gpc_norm1


def persistence_statistics(D):
    """
    Compute persistence statistics for a given persistence diagram.
    Args:
        D: persistence diagram in an n-by-2 array.
    Returns:
        persistence statistics, 10-dimensional vector.
    """
   ML = (D[:, 0] + D[:, 1])/2
   LS = D[:, 1] - D[:, 0]
   return np.array([np.mean(ML), np.std(ML), scipy.stats.kurtosis(ML), scipy.stats.skew(ML),
                               scipy.stats.entropy(np.abs(ML)),  np.mean(LS), np.std(LS),
                               scipy.stats.kurtosis(LS), scipy.stats.skew(LS),
                               scipy.stats.entropy(LS), get_GPC_norm1(D[:,:])] )
```

Listing 1: Sample code to compute TDA based features.

3 Feature Selection

To determine the number of features needed for the binary classifier, we randomly split the entire dataset (96 nights) as train, validation, and test sets with a 6-2-2 ratio. Elbow plots are generated from models with a different number of selected features with AUC reported on the test set. With 16 features in the accelerometer data model, the AUC can achieve 99.26% (0.812/0.818) of the AUC from the model with the full set of features (348). For the model with both accelerometer and gyroscope data, with 18 features, the AUC can achieve 99.05% (0.835/0.843) of the AUC from the model with the full set of features (686).

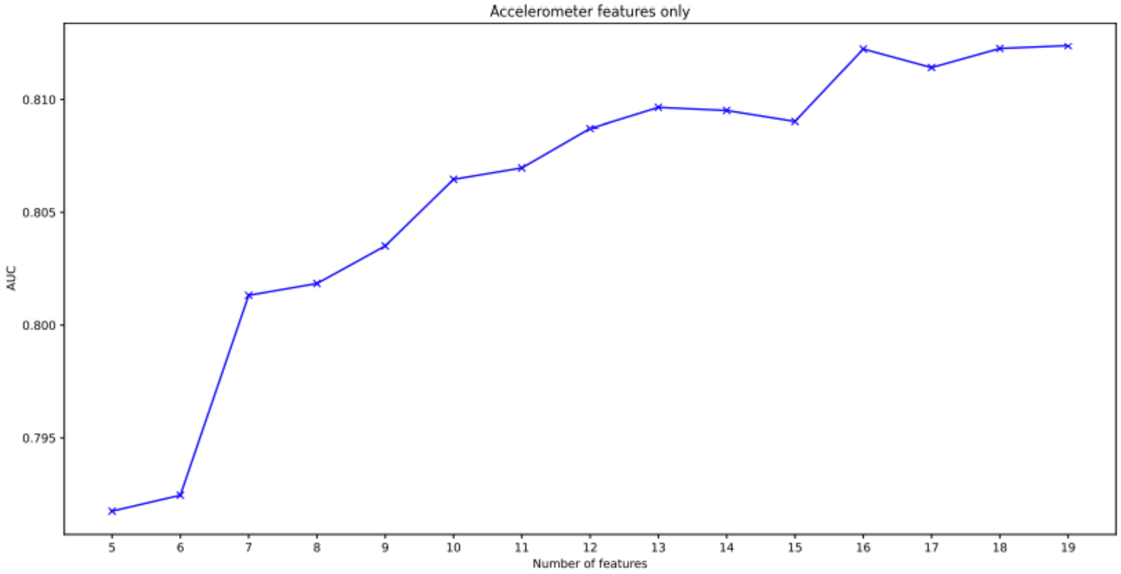

(a) Model with accelerometer data, sufficient number of features is 16

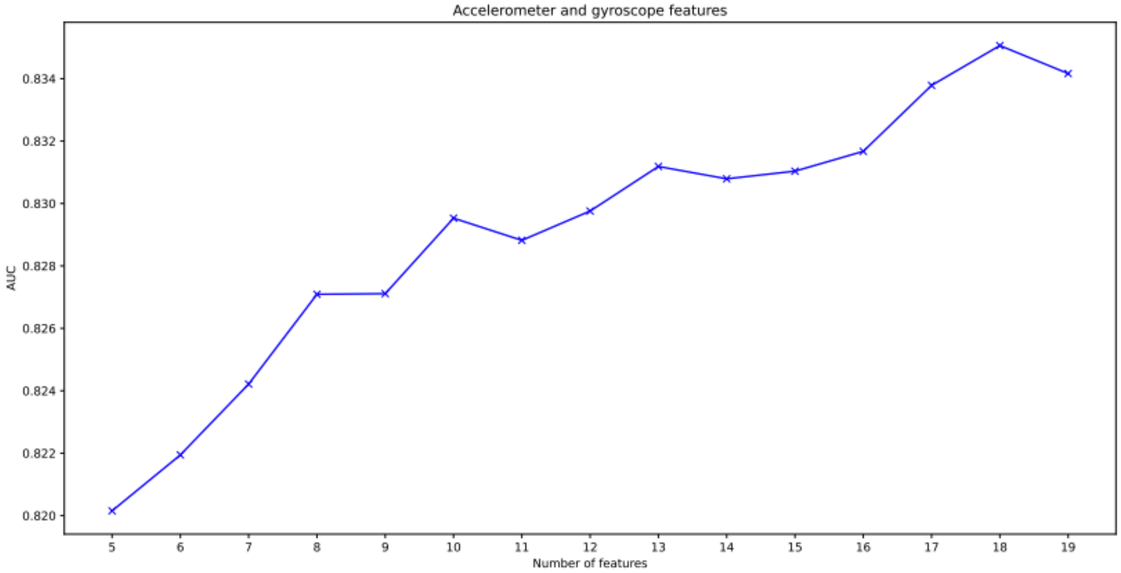

(b) Model with both accelerometer and gyroscope data, sufficient number of features is 18

Supplementary Figure S2: Elbow plots of feature selection.

4 Movement Algorithm and Scratch Detection Model Results

subject	# nights	proposed approach % scratch wrongly classified as non-movement	Mahadevan et al. % scratch wrongly classified as non-movement	proposed approach % prevalence	Mahadevan et al. % prevalence
1	5	1.383	12.648	4.556	5.587
2	5	12.070	43.395	31.290	28.266
3	5	3.425	28.220	45.968	50.290
4	5	3.892	17.216	16.713	20.206
5	5	8.763	29.897	36.152	43.889
6	5	2.019	10.978	29.526	34.258
7	5	2.252	11.768	36.611	43.418
8	5	4.905	24.124	40.038	43.806
9	5	9.703	47.596	16.320	17.469
10	5	5.244	28.659	7.632	9.279
11	4	16.367	32.044	10.216	10.887
12	5	7.319	20.189	11.307	12.909
13	5	8.496	25.152	13.884	17.919
14	5	6.124	19.823	8.511	9.422
15	5	6.173	25.365	9.398	15.937
16	5	16.244	30.047	5.581	6.112
17	2	46.243	53.179	2.023	2.503
18	5	7.077	19.040	6.131	7.588
19	5	7.798	20.154	15.428	16.294
20	5	18.026	37.707	9.443	9.478

Supplementary Table S1: Subject-level movement detection results.

subject	prevalence	AUC		accuracy		recall		specificity		F1		PPV		NPV	
		acc	acc &gyro	acc	acc &gyro	acc	acc &gyro	acc	acc &gyro	acc	acc &gyro	acc	acc &gyro	acc	acc &gyro
1	0.046	0.797	0.789	0.779	0.800	0.684	0.632	0.784	0.808	0.220	0.223	0.131	0.136	0.981	0.979
2	0.313	0.711	0.730	0.695	0.718	0.510	0.521	0.779	0.808	0.511	0.536	0.512	0.553	0.777	0.787
3	0.460	0.751	0.799	0.687	0.721	0.616	0.670	0.746	0.764	0.644	0.689	0.674	0.708	0.696	0.732
4	0.167	0.771	0.834	0.664	0.861	0.755	0.490	0.646	0.935	0.429	0.540	0.300	0.602	0.929	0.901
5	0.362	0.819	0.808	0.762	0.744	0.608	0.508	0.848	0.877	0.649	0.589	0.694	0.701	0.793	0.759
6	0.295	0.785	0.852	0.738	0.786	0.216	0.794	0.957	0.783	0.327	0.687	0.677	0.605	0.744	0.901
7	0.366	0.856	0.869	0.767	0.795	0.817	0.788	0.737	0.799	0.719	0.738	0.642	0.694	0.875	0.867
8	0.400	0.724	0.832	0.663	0.759	0.733	0.737	0.617	0.774	0.635	0.710	0.561	0.685	0.776	0.815
9	0.163	0.660	0.714	0.681	0.714	0.509	0.574	0.715	0.741	0.343	0.396	0.258	0.302	0.882	0.899
10	0.076	0.740	0.751	0.876	0.731	0.200	0.624	0.931	0.740	0.197	0.262	0.194	0.166	0.934	0.960
11	0.102	0.770	0.790	0.763	0.765	0.601	0.642	0.781	0.779	0.341	0.359	0.238	0.249	0.945	0.950
12	0.113	0.787	0.813	0.730	0.776	0.715	0.720	0.732	0.783	0.375	0.421	0.254	0.297	0.953	0.956
13	0.139	0.746	0.791	0.752	0.785	0.589	0.627	0.778	0.810	0.397	0.447	0.300	0.348	0.921	0.931
14	0.085	0.796	0.817	0.854	0.807	0.463	0.651	0.891	0.822	0.351	0.365	0.283	0.253	0.947	0.962
15	0.094	0.815	0.833	0.762	0.763	0.693	0.750	0.769	0.764	0.353	0.373	0.237	0.248	0.960	0.967
16	0.056	0.811	0.853	0.793	0.862	0.638	0.601	0.802	0.878	0.256	0.327	0.160	0.225	0.974	0.974
17	0.020	0.678	0.729	0.870	0.885	0.348	0.304	0.881	0.897	0.098	0.097	0.057	0.057	0.985	0.984
18	0.061	0.783	0.829	0.704	0.790	0.718	0.664	0.704	0.798	0.230	0.280	0.137	0.177	0.974	0.973
19	0.154	0.829	0.851	0.817	0.812	0.701	0.752	0.838	0.823	0.541	0.553	0.441	0.437	0.939	0.948
20	0.094	0.720	0.737	0.755	0.693	0.532	0.653	0.778	0.698	0.290	0.287	0.200	0.184	0.941	0.951

Supplementary Table S2: Leave-one-subject-out evaluation for binary scratch classifier

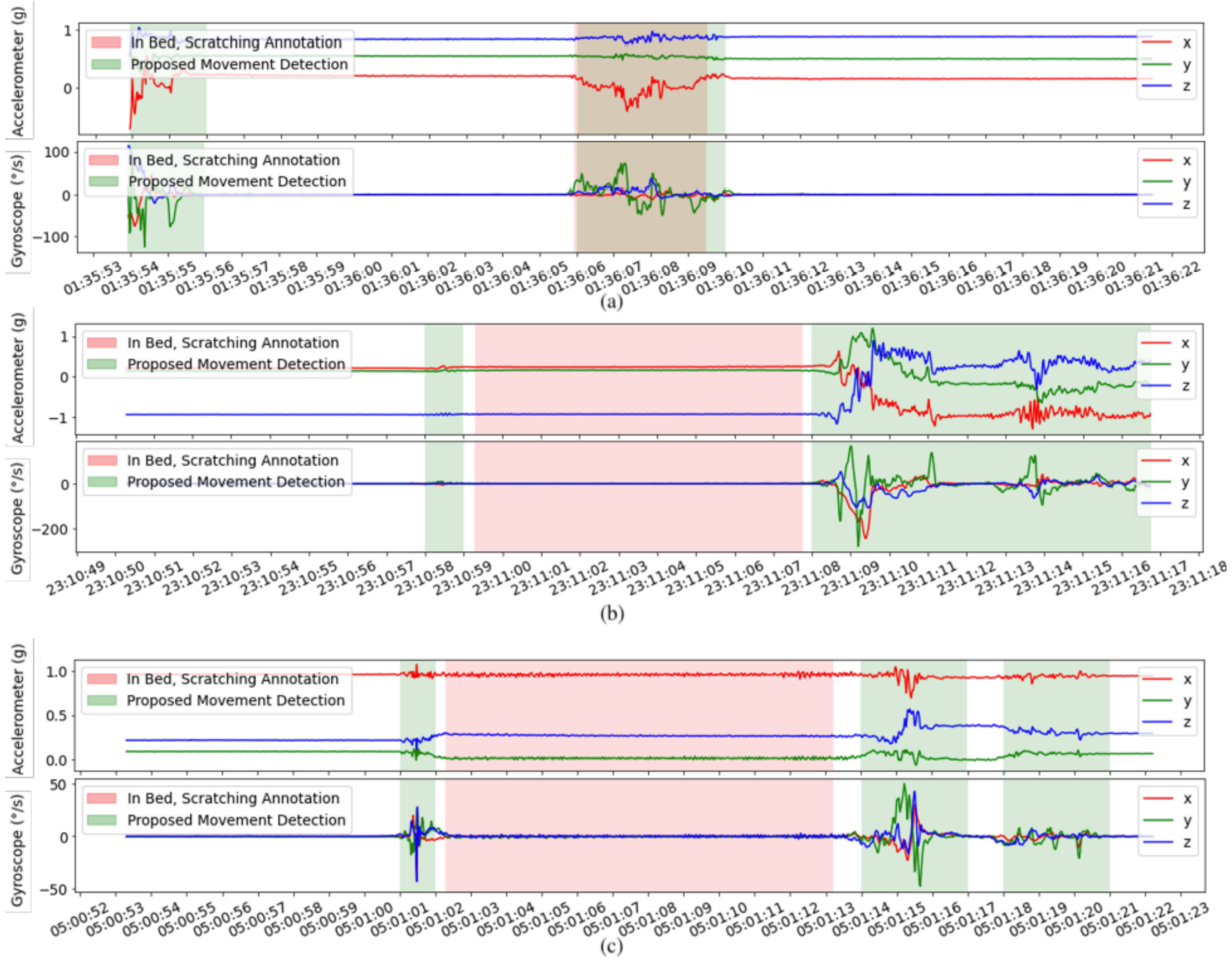

Supplementary Figure S3: Plots of raw accelerometer and gyroscope signals overlapped with video ground truth and movement detection results. (a) The algorithm correctly identifies an annotated scratching event as movement (red and green shades overlapped). (b) Annotated scratching event with flat signal and was not identified as movement by the algorithm (no overlap between red and green shades). (c) Annotated scratching event with slight noise and was not identified as movement by the algorithm (no overlap between red and green shades.

5 Distributions of ADSS and SCORAD

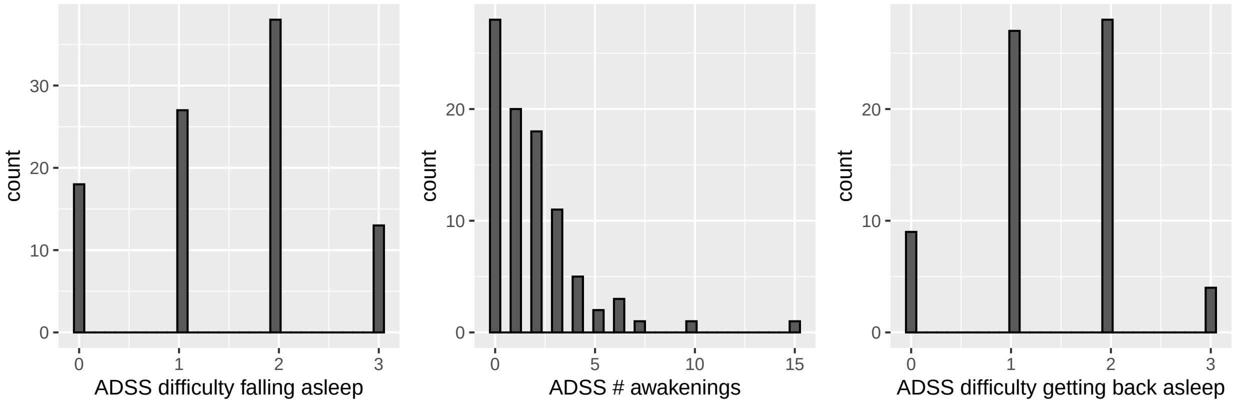

Supplementary Figure S4: Distribution of ADSS

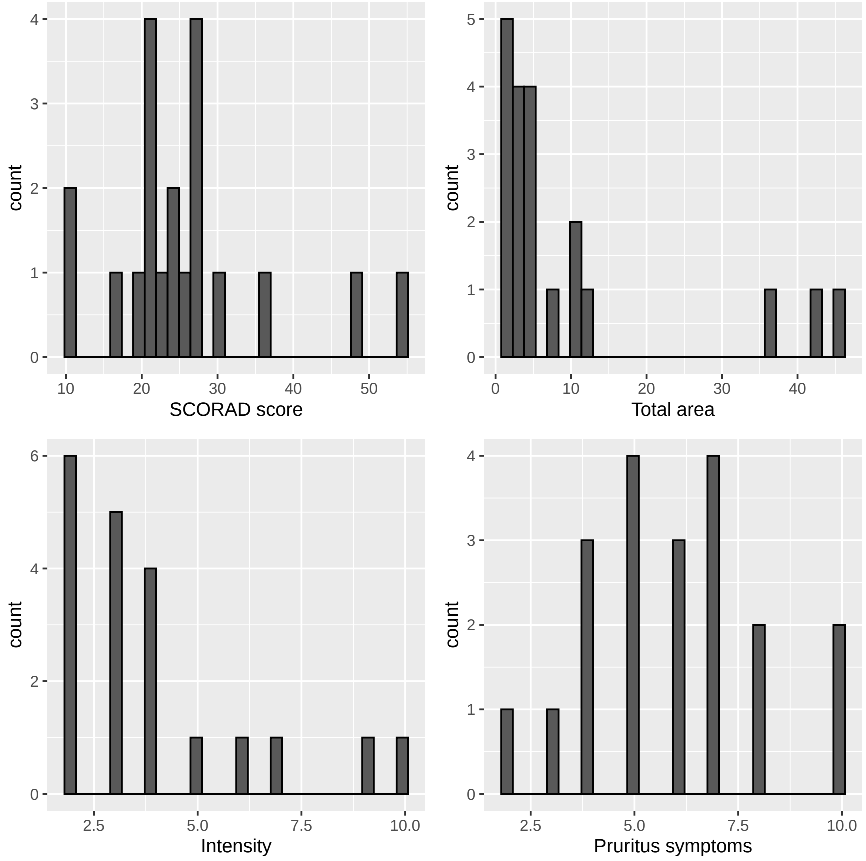

Supplementary Figure S5: Distribution of SCORAD

References

[1] Michael Boyle. The integration of angular velocity. *Advances in Applied Clifford Algebras*, 27(3):2345–2374, 2017.

[2] Peter Bubenik et al. Statistical topological data analysis using persistence landscapes. *J. Mach. Learn. Res.*, 16(1):77–102, 2015.

[3] Peter Bubenik and Alexander Wagner. Embeddings of persistence diagrams into hilbert spaces. *Journal of Applied and Computational Topology*, 4(3):339–351, 2020.

[4] Gunnar Carlsson. Topology and data. *Bulletin of the American Mathematical Society*, 46(2):255–308, 2009.

[5] Gunnar Carlsson and Mikael Vejdemo-Johansson. *Topological Data Analysis with Applications*. Cambridge University Press, 2021.

[6] Frédéric Chazal and Bertrand Michel. An introduction to topological data analysis: fundamental and practical aspects for data scientists. *arXiv preprint arXiv:1710.04019*, 2017.

[7] Yu-Min Chung, Chuan-Shen Hu, Yu-Lun Lo, and Hau-Tieng Wu. A persistent homology approach to heart rate variability analysis with an application to sleep-wake classification. *Frontiers in Physiology*, 12:202, 2021.

[8] Yu-Min Chung, Michael Hull, Austin Lawson, and Neil Pritchard. Gaussian persistence curves. *arXiv preprint arXiv:2205.11353*, 2022.

[9] Yu-Min Chung and Austin Lawson. Persistence curves: A canonical framework for summarizing persistence diagrams. *Advances in Computational Mathematics*, 48(1):1–42, 2022.

[10] Herbert Edelsbrunner and John Harer. *Computational topology: an introduction*. American Mathematical Soc., 2010.

[11] Robert Ghrist. Barcodes: the persistent topology of data. *Bulletin of the American Mathematical Society*, 45(1):61–75, 2008.

[12] Basile Graf. Quaternions and dynamics. *arXiv preprint arXiv:0811.2889*, 2008.

[13] Alperen Karan and Atabey Kaygun. Time series classification via topological data analysis. *Expert Systems with Applications*, page 115326, 2021.

[14] Austin Lawson, Yu-Min Chung, and William Cruse. A hybrid metric based on persistent homology and its application to signal classification. In *2020 25th International Conference on Pattern Recognition (ICPR)*, pages 9944–9950. IEEE, 2021.

[15] Yuriy Mileyko, Sayan Mukherjee, and John Harer. Probability measures on the space of persistence diagrams. *Inverse Problems*, 27(12):124007, 2011.

[16] Alice Patania, Francesco Vaccarino, and Giovanni Petri. Topological analysis of data. *EPJ Data Science*, 6:1–6, 2017.

[17] Yan Pei, Swarnendu Biswas, Donald S Fussell, and Keshav Pingali. An elementary introduction to kalman filtering. *Communications of the ACM*, 62(11):122–133, 2019.

[18] Jose A Perea, Anastasia Deckard, Steve B Haase, and John Harer. Sw1pers: Sliding windows and 1-persistence scoring; discovering periodicity in gene expression time series data. *BMC bioinformatics*, 16(1):1–12, 2015.

[19] Jose A Perea and John Harer. Sliding windows and persistence: An application of topological methods to signal analysis. *Foundations of Computational Mathematics*, 15(3):799–838, 2015.

[20] Nalini Ravishanker and Renjie Chen. An introduction to persistent homology for time series. *Wiley Interdisciplinary Reviews: Computational Statistics*, 13(3):e1548, 2021.

[21] The GUDHI Project. *GUDHI User and Reference Manual*. GUDHI Editorial Board, 3.4.1 edition, 2021.

[22] Yuhei Umeda. Time series classification via topological data analysis. *Information and Media Technologies*, 12:228–239, 2017.

[23] Larry Wasserman. Topological data analysis. *Annual Review of Statistics and Its Application*, 5:501–532, 2018.

[24] Afra Zomorodian. Topological data analysis. *Advances in applied and computational topology*, 70:1–39, 2012.
